# Genomic profiling of lower-grade gliomas uncovers cohesive disease groups: implications for diagnosis and treatment

**DOI:** 10.1186/s40880-015-0071-1

**Published:** 2016-01-12

**Authors:** Chang-Ming Zhang, Daniel J. Brat

**Affiliations:** Xiangya School of Medicine, Central South University, Changsha, 410008 Hunan P. R. China; Department of Pathology and Laboratory Medicine, Winship Cancer Institute, Emory University School of Medicine, Atlanta, GA 30322 USA

**Keywords:** Lower-grade glioma, The Cancer Genome Atlas, Histologic class, Molecular class, Isocitrate dehydrogenase (*IDH*) mutation

## Abstract

Lower-grade gliomas (including low- and intermediate-grade gliomas, World Health Organization grades II and III) are diffusely infiltrative neoplasms that arise most often in the cerebral hemispheres of adults and have traditionally been classified based on their presumed histogenesis as astrocytomas, oligodendrogliomas, or oligoastrocytomas. Although the histopathologic classification of lower-grade glioma has been the accepted standard for nearly a century, it suffers from high intra- and inter-observer variability and does not adequately predict clinical outcomes. Based on integrated analysis of multiplatform genomic data from The Cancer Genome Atlas, lower-grade gliomas have been found to segregate into three cohesive, clinically relevant molecular classes. Molecular classes were closely aligned with the status of isocitrate dehydrogenase (*IDH*) mutations, tumor protein 53 mutations and the co-deletion of chromosome arms 1p and 19q, but were not closely aligned with histologic classes. These findings emphasize the potential for improved definition of clinically relevant disease subsets using integrated molecular approaches and highlight the importance of biomarkers for brain tumor classification.

## Background

Diffuse low-grade and intermediate-grade gliomas [World Health Organization (WHO) grades II and III, hereafter called lower-grade gliomas] are infiltrative neoplasms that arise most often in the cerebral hemispheres of adults [1, 2]. Histologic criteria for the diagnosis of diffuse gliomas date from the early 20th century work of Bailey and Cushing [3], who grouped tumors by their presumed histogenesis based on their appearance under the microscope (i.e., classes were based on their “cell of origin”). Since then, histologic diagnosis has remained an efficient and cost-effective method for classification and grading, serving as the international standard [2]. Using these methods, lower-grade gliomas have traditionally been categorized into three classes: oligodendrogliomas, astrocytomas, and oligoastrocytomas. Following classification, diffuse gliomas are graded II–IV based on the presence of other morphologic features that reflect biological behavior, such as mitosis, microvascular proliferation, and necrosis, in order to predict clinical outcome and guide patient management. Despite widespread acceptance for nearly a century, it has been recognized that histologic classification and grading suffer from substantial inter- and intra-observer variability and do not adequately predict clinical outcomes [4, 5].

Increasingly, biomarker-based classification has been used to categorize disease and guide clinical decision making [6, 7]. For example, isocitrate dehydrogenase (*IDH*) mutations have been shown to be associated with a favorable prognosis in diffuse gliomas. The finding of co-deletion of chromosome arms 1p and 19q (1p/19q co-deletion) has been strongly linked with the oligodendroglioma histology and predicts better responses to radiochemotherapy. In order to determine if robust, clinically meaningful molecular classes of lower-grade gliomas could be identified and recognized by specific biomarkers, The Cancer Genome Atlas (TCGA) recently performed a comprehensive and integrated molecular analysis [8]. In their study, 293 untreated diffuse lower-grade glioma samples were collected from adults, including 100 astrocytomas, 77 oligoastrocytomas, and 116 oligodendrogliomas [8]. A comprehensive molecular analysis was performed for mutations using whole-exome sequencing, DNA copy number alterations, DNA methylation, microRNA expression, mRNA expression, and targeted protein expression. The results were integrated by two independent, multiplatform methods: cluster of clusters (CoC), which integrated data from gene expression (mRNA), DNA methylation, miRNA expression, and copy number alteration platforms; and OncoSign, which integrated mutations, amplifications, and deletions.

These two independent and unsupervised analyses each pointed to three robust molecular classes of lower-grade gliomas. Investigators then compared these three molecular classes to histologic classification of gliomas and to the biomarker classification of lower-grade glioma based on the status of *IDH* mutation and 1p/19q co-deletion. For both the CoC and OncoSign analyses, the association between molecular class and *IDH*–1p/19q co-deletion status was far superior to the association between molecular class and histologic class, indicating that these biomarkers were capable of accurately capturing cohesive biological characteristics of lower-grade gliomas in a robust manner. Based on these studies, lower-grade gliomas were designated as either (1) *IDH* mutant with 1p/19q co-deletion; (2) *IDH* mutant without 1p/19q co-deletion; or (3) *IDH* wild-type.

**Fig. 1:**
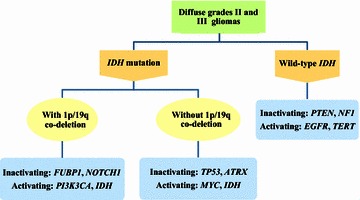
Schematic representation of diffuse glioma molecular classification. Lower-grade gliomas, the diffuse low-grade and intermediate-grade gliomas (World Health Organization grades II and III), are divided into three cohesive molecular subtypes based on molecular markers, including isocitrate dehydrogenase (*IDH*) mutations, co-deletion of chromosome arms 1p and 19q (1p/19q co-deletion), and tumor protein p53 (*TP53*)

The first subtype of lower-grade gliomas, characterized by both *IDH* mutations and 1p/19q co-deletion, showed a strong association with the oligodendroglioma histologic class, consistent with the results of numerous studies which emphasized that the combination of *IDH* mutation and 1p/19q co-deletion is the molecular signature of this disease (Fig. 1) [9]. Other findings in this subtype included activating mutations of the telomerase reverse transcriptase (*TERT*) promoter in nearly all cases (96%) [10]. Other frequent mutations were noted in capicua transcriptional repressor (*CIC*), neurogenic locus notch homolog protein 1 (*NOTCH1*), far upstream element-binding protein 1 (*FUBP1*), and the PI3 kinase pathway gene phosphatidylinositol-4, 5-bisphosphate 3-kinase, catalytic subunit alpha (*PIK3CA*). Of note, this subset was associated with the most favorable clinical outcomes, with a median patient survival of 8.0 years, partially due to the well-recognized radiochemosensitivity of tumors with 1p/19q co-deletion.

The second subtype of lower-grade gliomas had *IDH* mutations but lacked 1p/19q co-deletion and *TERT* promoter mutations (Fig. 1). Instead, they were characterized by mutations or losses of alpha thalassemia/mental retardation syndrome X-linked (*ATRX*) and tumor protein p53 (*TP53*) in 86 and 94% of cases, respectively. Thus, in *IDH*-mutant lower-grade gliomas, a strong, mutually exclusive relationship exists between *TP53* mutations and 1p/19q co-deletions. *IDH*-mutant lower-grade gliomas have either one or the other of these molecular events, but rarely both, indicating a strict molecular dichotomy. Lower-grade gliomas with *IDH*, *TP53*, and *ATRX* alterations have the molecular signature of diffuse astrocytoma and a median patient survival of 6.3 years [11].

The third molecular subtype of lower-grade gliomas had wild-type *IDH* and displayed a range of genetic alterations completely distinct from those of *IDH*-mutant lower-grade gliomas (Fig. 1). Genetic alterations in *IDH* wild-type tumors showed a remarkable resemblance to primary glioblastoma (WHO grade IV) across all analytic platforms [including genetic aberrations in phosphatase and tensin homolog (*PTEN*), neurofibromin *1* (*NF1*), cyclin-dependent kinase inhibitor 2A (*CDKN2A*), TP53 regulator (*MDM4*), *TERT* promoter, and epidermal growth factor receptor (*EGFR*)]. Both the genomic properties and clinical behavior suggested that *IDH* wild-type lower-grade gliomas are immediate precursors to *IDH* wide-type glioblastoma.

This unsupervised analysis of genome-wide molecular data identified three robust disease categories that can be recognized using well-established biomarkers, including *IDH*, 1p/19q, *TP53*, *ATRX*, and *TERT*. Importantly, these molecularly defined subsets are cohesive and reproducible, whereas histologic classes, by themselves, are not. For example, tumors diagnosed as “oligoastrocytoma” were found in all three molecular classes and lacked a defining molecular signature. Previously, diagnostic criteria for oligoastrocytomas have been inconsistent, and many lower-grade gliomas with “ambiguous morphology” have been treated as oligoastrocytomas [12]. Results indicated that lower-grade gliomas with an *IDH* mutation have either 1p/19q co-deletion or a *TP53* mutation, reflecting two distinct molecular mechanisms of oncogenesis; furthermore, no evidence exists for a biological or genetic signature specific to oligoastrocytoma. Based on these findings and others, molecular markers can be used to determine lineage rather than histologic appearance, and the proportion of “oligoastrocytoma” diagnosis among lower-grade gliomas will likely decrease.

Ultimately, the studies by TCGA indicate that advances in molecular profiling will allow neoplastic diseases to be defined using a new approach. Unsupervised clustering of whole-genome molecular data has ushered in a new age in which biological classes of disease can be precisely identified. In the case of lower-grade gliomas, well-established genetic markers (such as *IDH*, 1p/19q, *ATRX*, *TP53*, and *TERT*) were capable of identifying new disease classes with high fidelity. In histologic classification, ambiguous morphology is common, resulting in low reproducibility and inter-observer concordance, leading to confusion in clinical management. Molecular classification represents an improvement in diagnostic practice, enabling practitioners to identify clinically distinct neoplasms and confidently direct appropriate standard therapies or clinical trials.

## Abbreviations

*PTEN*: phosphatase and tensin homolog
*NF1*: neurofibromin 1
*EGFR*: epidermal growth factor receptor
*TERT*: telomerase reverse transcriptase
*FUBP1*: far upstream element-binding protein 1
*NOTCH1*: neurogenic locus notch homolog protein 1
*PI3K3CA*: the PI3 kinase pathway gene phosphatidylinositol-4,5-bisphosphate 3-kinase, catalytic subunit alpha
*TP53*: tumor protein 53
*ATRX*: alpha thalassemia/mental retardation syndrome X-linked
*MYC*: v-myc avian myelocytomatosis viral oncogene homolog

## References

[1] Ostrom QT, Gittleman H, Farah P, Ondracek A, Chen Y, Wolinsky Y (2013). CBTRUS statistical report: primary brain and central nervous system tumors diagnosed in the United States in 2006–2010. Neuro Oncol.

[2] Louis DN, Ohgaki H, Wiestler OD, Cavenee WK, Burger PC, Jouvet A (2007). The 2007 WHO classification of tumours of the central nervous system. Acta Neuropathol.

[3] Bailey H (1926). The abdominal crises of pernicious anaemia. Br Med J.

[4] Coons SW, Johnson PC, Scheithauer BW, Yates AJ, Pearl DK (1997). Improving diagnostic accuracy and interobserver concordance in the classification and grading of primary gliomas. Cancer.

[5] van den Bent MJ (2010). Interobserver variation of the histopathological diagnosis in clinical trials on glioma: a clinician’s perspective. Acta Neuropathol.

[6] Brat DJ, Cagle PT, Dillon DA, Hattab EM, McLendon RE, Miller MA (2015). Template for reporting results of biomarker testing of specimens from patients with tumors of the central nervous system. Arch Pathol Lab Med.

[7] Appin CL, Brat DJ (2015). Biomarker-driven diagnosis of diffuse gliomas. Mol Aspects Med.

[8] Network Cancer Genome Atlas Research, Brat DJ, Verhaak RG, Aldape KD, Yung WK, Salama SR (2015). Comprehensive, integrative genomic analysis of diffuse lower-grade gliomas. N Engl J Med.

[9] Yip S, Butterfield YS, Morozova O, Chittaranjan S, Blough MD, An J (2012). Concurrent CIC mutations, IDH mutations, and 1p/19q loss distinguish oligodendrogliomas from other cancers. J Pathol.

[10] Eckel-Passow JE, Lachance DH, Molinaro AM, Walsh KM, Decker PA, Sicotte H (2015). Glioma groups based on 1p/19q, IDH, and TERT promoter mutations in tumors. N Engl J Med.

[11] Jiao Y, Killela PJ, Reitman ZJ, Rasheed AB, Heaphy CM, de Wilde RF (2012). Frequent ATRX, CIC, FUBP1 and IDH1 mutations refine the classification of malignant gliomas. Oncotarget.

[12] Sahm F, Reuss D, Koelsche C, Capper D, Schittenhelm J, Heim S (2014). Farewell to oligoastrocytoma: in situ molecular genetics favor classification as either oligodendroglioma or astrocytoma. Acta Neuropathol.

